# Supplementary material for: Detection of Germline Variants in 450 Breast/Ovarian Cancer Families with a Multi-Gene Panel Including Coding and Regulatory Regions
Source: Int J Mol Sci. 2021 Jul 19;22(14):7693. doi: 10.3390/ijms22147693 (PMC8305371; doi:10.3390/ijms22147693)
Supplement: Supplementary file 1 [file ijms-22-07693-s001.zip › ijms-1277723-supplementary.pdf]

Gene	Nt.Change	Function	Clinical Classification	Position	N.Patient	PolyPhen2(HumDiv)	PROVEAN	Database	dbSNP
ATM	c.1810C>T	Missense	Likely Benign	exon 12	2	POSSIBLY DAMAGING	Neutral (-2.165)	LOVD	rs2227922
ATM	c.6067G>A	Missense	Pathogenic	exon 41	1	PROBABLY DAMAGING	Deleterious (-3.494)	LOVD	rs11212587
ATM	c.998C>T	Missense	Uncertain Significance	exon 8	1	POSSIBLY DAMAGING	Neutral (-1.592)	LOVD	rs28904919
ATM	c.2254C>G	Missense	Uncertain Significance	exon 15	1	PROBABLY DAMAGING	Neutral (-1.168)		rs756522395
ATM	c.5611A>C	Missense	Uncertain Significance	exon 37	1	PROBABLY DAMAGING	Neutral (-2.327)		rs990967899
ATM	c.4060C>A	Missense	Uncertain Significance	exon 27	2	BENIGN	Neutral (-2.013)	LOVD	rs145119475
ATM	c.4703A>G	Missense	Uncertain Significance	exon 31	1	BENIGN	Neutral (-1.432)	LOVD	rs368830730
ATM	c.6293T>C	Missense	Likely Pathogenic	exon 43	1	PROBABLY DAMAGING	Deleterious (-3.669)	MGeND	rs587780631
ATM	c.9023G>A	Missense	Pathogenic	exon 63	1	PROBABLY DAMAGING	Deleterious (-4.004)	LOVD	rs587781894
ATM	c.2317A>G	Missense	Uncertain Significance	exon 15	1	BENIGN	Neutral (-0.365)		
ATM	c.7778A>G	Missense	Uncertain Significance	exon 52	1	BENIGN	Neutral (-1.424)	MGeND	rs587779867
ATM	c.5558A>T	Missense	Likely Benign	Exon 37	1	POSSIBLY DAMAGING	Deleterious (-4.519)	LOVD	rs1801673
ATM	c.2572T>C	Missense	Likely Benign	exon 17	1	POSSIBLY DAMAGING	Deleterious (-2.574)	LOVD	rs1800056
ATM	c.1960C>A	Missense	Uncertain Significance	exon 13	1	BENIGN	Neutral (-0.406)	LOVD	rs528165789
ATM	c.2119T>C	Missense	Likely Benign	exon 13	2	BENIGN	Neutral (-0.383)	LOVD	rs4986761|
ATM	c.1272T>C	Synonymous	Uncertain Significance	exon 10	1		Neutral (0)	LOVD	rs35578748

ATM	c.3894dupT	Frameshift	Pathogenic	exon 26	3			ClinVar Miner	rs587781823
ATM	c.7382G>A	Missense	Likely Pathogenic	exon 50	1	PROBABLY DAMAGING	Neutral -1748	LOVD	rs768461085
ATM	c.1601 C>G	Missense	Uncertain Significance	exon 10	2	PROBABLY DAMAGING	Deleterious -4.338	LOVD	rs587782212
ATM	c.7456C>T	Nonsense	Pathogenic	exon 50	1			LOVD	rs587779865
ATM	c.2945G>A	Missense	Uncertain Significance	exon 20	1	POSSIBLY DAMAGING	Neutral -1.741		rs749471737
ATM	c.1516G>T	Missense	Uncertain Significance	exon 10	1	POSSIBLY DAMAGING	Neutral -2.030	LOVD	rs587779816
ATM	c.1700A>G	Missense	Uncertain Significance	exon 11	1	BENIGN	Neutral -0.861		rs786203230
ATM	c.1595G>A	Missense	Uncertain Significance	exon 10	1	PROBABLY DAMAGING	Deleterious -3839	LOVD	rs35963548
ATM	c.5897G>A	Missense	Uncertain Significance	exon 39	1	BENIGN	Neutral 0.133	ClinVar Miner	rs1555110520
ATM	c.8122G>A	Missense	Pathogenic	exon 55	1	PROBABLY DAMAGING	Deleterious -4.148	LOVD	rs587782719
ATM	c.7269A>T	Missense	Likely Pathogenic	exon 49	1	PROBABLY DAMAGING	Neutral -1.250		rs864622471
ATM	c.8083G>A	Missense	Likely Pathogenic	exon 55	1	PROBABLY DAMAGING	Deleterious -5177	LOVD	rs1555127166
ATM	c.8368delA	Frameshift	Pathogenic	exon 57	1				
ATM	c.6703A>G	Missense	Uncertain Significance	exon 46	1	BENIGN	Neutral -0.351		rs768791795
ATM	c.146C>T	Missense	Uncertain Significance	exon 3	1	POSSIBLY DAMAGING	Neutral -2.073	LOVD	rs1800054
ATM	c.8734A>G	Missense	Likely Pathogenic	exon 60	1	PROBABLY DAMAGING	Deleterious -5833	LOVD	rs376676328
ATM	c.3284G>A	Missense	Likely Pathogenic	exon 22	1	POSSIBLY DAMAGING	Neutral -0.238	LOVD	rs587781815
ATM	c.7671_7674delGTTT	Frameshift	Pathogenic	exon 52	1				rs1555124506

ATM	c.6226A>G	Missense	Uncertain Significance	exon 43	1	POSSIBLY DAMAGING	Neutral (-0.285)		rs755973863
ATM	c.7475T>G	Missense	Likely Pathogenic	exon 50	1	PROBABLY DAMAGING	Deleterious (-4.533)	LOVD	rs56399857
BRIP1	c.2392C>T	Nonsense	Pathogenic	exon 17	1			LOVD	rs137852986
BRIP1	c.139C>G	Missense	Pathogenic	exon 3	2	PROBABLY DAMAGING	Deleterious (-6.458)	LOVD	rs28903098
BRIP1	c.2448G>T	Missense	Uncertain Significance	exon 17	1	PROBABLY DAMAGING	Deleterious (-12.28)		rs1064795352
BRIP1	c.316C>T	Missense	Uncertain Significance	exon 4	1	BENIGN	Neutral (-0.36)	MGeND	rs587780247
BRIP1	c.2220G>T	Missense	Likely Benign	exon 15	1	PROBABLY DAMAGING	Neutral (-2.20)	LOVD	rs45589637
BRIP1	c.2325T>G	Missense	Uncertain Significance	exon 16	1	PROBABLY DAMAGING	Deleterious (-4.45)		rs375146450
BRIP1	c.3029A>G	Missense	Uncertain Significance	exon 20	1	BENIGN	Neutral (-0.32)		
BRIP1	c.2237_2240delTCAA	Frameshift	Pathogenic	exon 15	1			MGeND	rs587782726
BRIP1	c.1201_1204dupTGTG	Frameshift	Pathogenic	exon 9	2				rs730881647
BRIP1	c.582A>T	Missense	Uncertain Significance	exon 6	1	PROBABLY DAMAGING	Neutral (-1.05)		rs796681126
BRIP1	c.2992_2995delAAGA	Frameshift	Pathogenic	exon 20	1				rs786203717
CDH1	c.2074G>A	Missense	Likely Benign	exon 13	1	POSSIBLY DAMAGING	Neutral (-0.776)	ClinVar Miner	rs376854556
CDH1	c.2413G>A	Missense	Likely Benign	exon 15	1	PROBABLY DAMAGING	Deleterious (-2.633)	LOVD	rs200894246
CDH1	c.674T>C	Missense	Uncertain Significance	exon 5	3	PROBABLY DAMAGING	Deleterious (-2.67)	MGeND	rs786203207
CDH1	c.131G>T	Missense	Likely Benign	exon 2	1	BENIGN	Neutral (-1.88)		rs1375178645
CDH1	c.2623A>G	Missense	Uncertain Significance	exon 16	1	POSSIBLY DAMAGING	Deleterious (-2.84)		rs1555518287
CHEK2	c.1312G>T	Missense	Likely Pathogenic	exon 13	1	PROBABLY DAMAGING	Deleterious (-4.888)	LOVD	rs587780170

CHEK2	c.483_485delAGA	Inframe deletion	Likely Pathogenic	exon 4	1		Deleterious(-10.48)	MGeND	rs587782008
CHEK2	c.507delT	Frameshift	Likely Pathogenic	exon 4	1				
CHEK2	c.1441G>T	Missense	Uncertain Significance	exon 13	1	PROBABLY DAMAGING	Deleterious (-5.15)	LOVD	rs200050883
CHEK2	c.917G>C	Missense	Likely Pathogenic	exon 10	1	PROBABLY DAMAGING	Deleterious (-5.38)	LOVD	rs587780192
CHEK2	c.1430delC	Frameshift	Likely Pathogenic	exon 13	1				
CHEK2	c.132C>T	Synonymous	Likely Benign	exon 2	2		Neutral (0.00)	ClinVar Miner	rs199715101
CHEK2	c.409C>T	Nonsense	Pathogenic	exon 3	1			LOVD	rs730881701
CHEK2	c.1118A>G	Missense	Likely Pathogenic	exon 9	1	PROBABLY DAMAGING	Deleterious (-3.74)		rs894075046
CHEK2	c.911T>C	Missense	Likely Pathogenic	exon 9	1	PROBABLY DAMAGING	Deleterious (-3.58)		rs587782033
NBN	c.671G>A	Missense	Uncertain Significance	exon 6	1	POSSIBLY DAMAGING	Deleterious (-4.8)		rs199845467
NBN	c.643C>T	Missense	Uncertain Significance	exon 6	1	PROBABLY DAMAGING	Deleterious (-4.100)	LOVD	rs34767364
NBN	c.511A>G	Missense	Uncertain Significance	exon 5	1	PROBABLY DAMAGING	Neutral (-0.795)	LOVD	rs61754966
PALB2	c.1451T>A	Nonsense	Pathogenic	exon 4	1		Deleterious (-1642.549)	ClinVar Miner	rs786203714
PALB2	c.2506G>A	Missense	Uncertain Significance	exon 5	1	BENIGN	Neutral (-0.37)	LOVD	rs536644825
PALB2	c.2920_2921delAA	Frameshift	Pathogenic	exon 9	1				rs180177126
PALB2	c.2066C>T	Missense	Uncertain Significance	exon 5	1	BENIGN	Neutral (1.37)	LOVD	rs749602688
PALB2	c.1463G>C	Missense	Uncertain Significance	exon 4	1	BENIGN	Neutral (-1.52)	ClinVar Miner	rs76792659
PALB2	c.1565dupC	Frameshift	Likely Pathogenic	exon 4	1				

PTEN	c.350A>G	Missense	Uncertain Significance	exon 5	1	BENIGN	Neutral (-1.199)	LOVD	rs551221430
PTEN	c.17_18insGCG	Inframe Insertion	Uncertain Significance	exon 1	1		Neutral (0.00)		
RAD50	c.980G>A	Missense	Likely Benign	exon 7	1	PROBABLY DAMAGING	Neutral (-2.052)	LOVD	rs28903091
RAD50	c.1094G>A	Missense	Uncertain Significance	exon 8	1	BENIGN	Neutral (-0.171)	ClinVar Miner, MGeND	rs146370443
RAD50	c.1277A>G	Missense	Uncertain Significance	exon 9	1	PROBABLY DAMAGING	Deleterious (-2.772)	ClinVar Mider	rs145428112
RAD50	c.379G>A	Missense	Likely Benign	exon 4	1	PROBABLY DAMAGING	Neutral (-0.818)	LOVD	rs28903086
RAD51C	c.376G>A	Missense	Likely Benign	exon 2	1	BENIGN	Neutral (-1.771)	LOVD	rs61758784
RAD51C	c.414G>C	Missense	Pathogenic	exon 3	1	POSSIBLY DAMAGING	Deleterious (-3.6)	LOVD	rs267606999
RAD51C	c.397_398delCA	Frameshift	Pathogenic	exon 2	1				
RAD51D	c.26G>C	Missense	Likely Pathogenic	exon 1	1	POSSIBLY DAMAGING	Deleterious (-6.191)	LOVD	rs140825795
RAD51D	c.167T>G	Missense	Likely Benign	exon 2	1	POSSIBLY DAMAGING	Deleterious (-2.976)		rs745307359
RAD51D	c.252T>C	Synonymous	Likely Benign	exon 4	1		Neutral (0.00)		
RAD51D	c.735C>A	Synonymous	Likely Benign	exon 8	1		Neutral (0.00)		rs760789270
RAD51D	c.694C>T	Nonsense	Pathogenic	exon 8	1			LOVD	rs587780104
STK11	c.1211C>T	Missense	Likely Benign	exon 9	1	BENIGN	Neutral (-1.747)	LOVD	rs200078204
STK11	c.1273C>A	Missense	Uncertain Significance	exon 9	1	POSSIBLY DAMAGING	Neutral (-1.35)	LOVD	rs754853898
TP53	c.817C>G	Missense	Pathogenic	exon 8	1	PROBABLY DAMAGING	Deleterious (-6.693)	LOVD, IARC	rs121913343

Table S1: List of 89 coding variants identified in 450 of patients. The table reports the nucleotide change for each variant, their clinical classification (P = Pathogenic, LP = Likely-pathogenic, VUS = Variant of Unknown clinical Significance, LB = Likely-benign), their frequency identified in these patients, the predictive analysis on the effect of amino acid change on the protein function obtained by *Polyphen2* and *PROVEAN* software(cutoff=-2.5), whether the variants have been previously recorded in databases and their dbSNP code**.**

a)

Gene	Nt.change	Clinical Classification	Position	N.Patient	Database	Transcription factor	rVarBase/ENCODE	dbSNP
ATM	c.-24A>C	Uncertain Significance	5'UTR	1		GRE, GFI1, GATA1/2, OCT1, CREB, PADS, USF(Tfbind)	TF binding region Chromatin interaction	
BRCA1	c.-676 T>A	Uncertain Significance	5' UTR	1	BRCA exchange/LOVD	CEBP, Elk-1, NF-AT1, STAT4, HMG, STAT1 beta, UNC-86, STAT5a, AGL3, IRF-3, Pax4a, POU3F2(Promo), CREL(TFbind)	Bivalent/Poised TSS, TF binding region	rs8176072
BRCA1	c.-408T>A	Uncertain Significance	5' UTR	1	BRCA exchange/LOVD	CHOP, CEBPB, OCT1, HLF (Tfbind)	TF binding region,Chromatin interaction, lncRNA Bivalent/Poised Active TSS Flanking Active TSS	
BRCA2	c.-1193C>T	Uncertain Significance	5'UTR	1	BRCAexchange/LOVD	NRF2, GATA1/2, USF, T3R, Myod (Tfbind)	TF binding region Chromatin interaction	
CDH1	c.-54 G>C	Benign	5'UTR	1	LOVD	CAP, SP1, CP2 (Tfbind)	Chromatin interactive region	rs5030874
CDH1	c.-44G>A	Uncertain Significance	5'UTR	1		SP1, AP2, CREB(Tfbind)	TF binding region Chromatin interaction	rs886041159
PALB2	c.-138G>C	Uncertain Significance	5' UTR	1		ELK1, CETS1 (Promo,TFbind), CAP, NRF2 (TFbind), STAT4 (PROMO), pu.1 (Lasagna)	TF binding region, Chromatin interactive region, Bivalent/Poised TSS	rs552824227

PTEN	c.-944C>T	Uncertain Significance	5'UTR	1		ZF5, EF2(Promo), WT1-KT5(Lasagna), AP4, AP2, SP1, EGR1, GC (Tfbind)	Bivalent/Poised TSS, TF binding region, Chromatin interaction	
PTEN	c.-102A>C	Uncertain Significance	5'UTR	1		CAP (Tfbind)	TF binding region;CpG island;Chromatin interaction,Bivalent/Poised TSS<br>ZNF genes & repeats<br>Active TSS	
PTEN	c.-1027C>A	Uncertain Significance	5'UTR	1		SP1, AP2, MZF1 (TFbind)	TF binding region, CpG island, Chromatin interactive region, Enhancers, Bivalent/Poised TSS	
PTEN	c.-323C>T	Uncertain Significance	5'UTR	1		VMYB, ZIP (Tfbind)	TF binding region, CpG island, Chromatin interactive region, Enhancers, Bivalent/Poised TSS	
RAD51C	c.-15G>A	Uncertain Significance	5'UTR	1		T3R-beta1, RXR-alpha, USF(Tfbind)		
RAD51C	c.-395T>G	Uncertain Significance	5'UTR	1		OCT1, CDPCR1, CDPCR3HD, NKX25 (Tfbind), GR-beta (Promo)	TF binding region, CpG island, Chromatin interactive region	rs568361156
RAD51C	c.-1G>A	Uncertain Significance	5'UTR	1		PAX5, AP4, OCT1(Tfbind)	TF binding region<br>CpG island<br>Chromatin interaction	rs750045091
RAD51D	c.-116C>T	Uncertain Significance	5'UTR	1		HNF4, ARP1, COUP, AP2, VMYB(Tfbind), NF1(Tfbind, Promo), p53 (Promo), COUP TF2 (Lasagna, Tfbind)	TF binding region, Chromatin interactive region	
RAD51D	c.-239C>T	Likely Benign	5'UTR	1		ELK1, NRF2, AP4 (Tfbind), GCF, E2F1 (Promo), c-Myb(L)	TF binding region, Chromatin interactive region, Bivalent/Poised TSS	rs550346401

STK11	c.-636C>T	Uncertain Significance	5'UTR	1		STAT3, CREB, NFKB50, SP1(Tfbind)		rs981125953
STK11	c.-127T> C	Benign	5'UTR	1		Pax5, Solf1, AP2, E2 (Tfbind)	TF binding region, Chromatin interactive region	rs532196225

b)

Gene	Nt.change	Clinical Classification	Position	N.Patient	Database	rVarBase/ENCODE	TargetScanHuman/ miRDB	dbSNP
ATM	c.*2842A>G	Uncertain Significance	3'UTR	1		Enhancers, Genic enhancers	hsa-miR-7159-3p, hsa-miR-2117 (miRDB), hsa-miR-4328 (TSH)	rs186644530
ATM	c.*636A>G	Uncertain Significance	3'UTR	1		Chromatin interaction	hsa-miR-802 , hsa-miR-196a-1-3p (miRDB)hsa-miR-4704-5p (TSH)	rs1024163177
ATM	c.*26A>G	Uncertain Significance	3'UTR	1		Chromatin interaction	hsa-miR-5681a, hsa-miR-488-3p (miRDB- TSH)	rs1247328981
ATM	c.*537A>C	Uncertain Significance	3'UTR	1		Chromatin interaction		rs910689990
ATM	c.*811T>G	Uncertain Significance	3'UTR	1		Chromatin interaction	hsa-miR-3185(TSH)	rs748879858
BRCA1	c.*750A>G	Likely Benign	3'UTR	2	BRCAexchange	Enhancers		rs138782023
NBN	c.*724dupT	Uncertain Significance	3'UTR	1		Chromatin interactive region		rs564878448
PTEN	c.*353C>T	Benign	3' UTR	1	MGeND		hsa-miR-542-3p (seed region, TSH, miRDB)	rs181234898

PTEN	c.*1122_*1123insT	Uncertain Significance	3' UTR	1		TF binding region, Chromatin interaction		
PTEN	c.*4394T>A	Benign	3'UTR	1			miR-499a-5p (TSH)	rs527703056
PTEN	c.*923T>A	Uncertain Significance	3'UTR	1		TF binding region<br>Chromatin interaction<br>miRNA targets site	miR-193-3p (TSH)	rs1217913921
PTEN	c.*1152C>T	Benign	3'UTR	1		miRNA target site		rs552354954
PTEN	c.*2985_*2986delGT	Uncertain Significance	3'UTR	1		TF binding region<br>Chromatin interactive region		
PTEN	c.*4606_*4609delGCTT	Benign	3'UTR	1		Chromatin interaction	hsa-miR-616-5p, hsa-miR-373-5p, hsa-miR-371b-5p(miRDB)	rs754321761
RAD50	c.*1674A>G	Uncertain Significance	3'UTR	1		Chromatin interaction	hsa-miR-4307 (seed region), hsa-miR-4251, hsa-miR-539-5p, hsa-miR-33a-3p (seed region-TSH)	
RAD50	c.*134T>A	Uncertain Significance	3'UTR	1		Chromatin interaction	miR-6800-3p (TSH)	
RAD50	c.*114A>G	Uncertain Significance	3'UTR	1		Chromatin interaction	hsa-miR-4326, hsa-miR-4305(TSH)	
RAD50	c.*263G>A	Uncertain Significance	3'UTR	1		Chromatin interactive region	hsa-miR-4638, hsa-miR-1307(TSH, seed region), hsa-miR-11400(miRDB, seed region)	rs181991619
RAD51D	c.*106G>A	Likely Benign	3'UTR	1	LOVD/MgeND	Chromatin interactive region	hsa-miR-4324 (miRDB), hsa-miR-589-5p, hsa-miR-146a/b-5p, hsa-miR-7153-5p (TSH)	rs45494001
STK11	c.*261C>T	Uncertain Significance	3'UTR	1	ClinVar Miner	TF binding region<br>Chromatin interaction	hsa-miR-4707-3p, hsa-miR-662	rs1054219411

TP53	c.*409C>A	Benign	3'UTR	1		Chromatin interactive region	hsa-miR-11400, hsa-miR-4500, miR-98-5p, hsa-let-7a-5p (b, c, d, e, f, g, i)	rs191918079

c)

Gene	Nt.change	Clinical Classification	Position	N.Patient	Database	rVarBase/ENCODE	HumanSpicingFinder	dbSNP
ATM	c.7788+8G>T	Uncertain Significance	intron 52 splice site	1	LOVD		Alteration of an exonic ESE site.Potential alteration of splicing.	rs112775908
ATM	c.3154-4G>A	Uncertain Significance	intron 21 splice site	1	LOVD	Chromatin interactive region	No significant splicing motif alteration detected. This mutation has probably no impact on splicing.	rs199543313
ATM	c.2376+3A>G	Uncertain Significance	intron 15 splice site	1	LOVD	Chromatin interaction	No significant splicing motif alteration detected. This mutation has probably no impact on splicing.	rs758083563
BRIP1	c.1341-7A>C	Uncertain Significance	intron 9 splice site	1		TF binding region Chromatin interaction	No significant splicing motif alteration detected. This mutation has probably no impact on splicing.	
CHEK2	c.320-5T>C	Uncertain Significance	Intron 2 spice site	10	LOVD	Chromatin interaction	No significant splicing motif alteration detected. This mutation has probably no impact on splicing.	
RAD51C	c.904+5G>T	Pathogenic	intron 6 splice site	2	LOVD	Chromatin interaction	Chromatin interaction ,Alteration of the WT donor site,most probably affecting splicing.	rs587782702

d)

Gene	Nt.change	Clinical Classification	Position	N.Patient	Database	Transcription factor	rVarBase/ENCODE	dbSNP
ATM	c.560+19_560+22delTACT	Uncertain Significance	intron 6	1			Enhancers	rs748598016
ATM	c.2250+22A>C	Benign	Intron 14	1	LOVD	CP2 , P300,UBX, CRX, CDX1 , IRF-7A	Chromatin interactive region	rs3218692
BRCA1	c.81-3510_81-3505delCTTTTT	Uncertain Significance	intron 2	1		EVI1,SRY	TF binding region, Chromatin interaction,Enhancers, Flanking Active TSS Strong transcription	rs1428246728
BRCA1	c.81-3790G>C	Uncertain Significance	Intron 2	1	BRCA exchange	NF-IL6-2	Genic enhancers, Flanking Active TSS	rs193150833
BRCA1	c.81-4100A>C	Uncertain Significance	Intron 2	1			Genic enhancers, ZNF genes & repeats	rs771237151
BRCA1	c.80+247C>T	Uncertain Significance	Intron 2	1	BRCA exchange			rs921607280
BRCA1	c.5137+51_5137+52delAT	Uncertain Significance	Intron 17	1	BRCAexchange	AP-2alphaA , PADS, MYOD, AP4, USF, AML1, C/EBP		rs1212800520
BRCA2	c.9256+4820C>T	Uncertain Significance	intron 24	2	BRCA exchange		New Donor Site-Activation of an intronic cryptic donor site.Potential alteration of splicing. (HSF)	rs1196532981
BRCA2	c.9256+1464C>T	Uncertain Significance	Intron 24	1	BRCA exchange		TF binding region, Chromatin interaction	rs945273697
BRCA2	c.9257-3610G>A	Uncertain Significance	intron24	1	BRCAexchange		Enhancers	rs17077519
BRCA2	c.681+697C>T	Uncertain Significance	intron 8	1	BRCAexchange		Enhancers, Genic enhancers	rs11571632

BRCA2	c.317-1021A>G	Uncertain Significance	intron 3	2	BRCAexchange	CDPCR1, HNF3B, CAP (TFBIND), GR-beta, C/EBPalpha (Promo)	Activation of an intronic cryptic acceptor/donor site,Potential alteration of splicing.(HSF)Enhancers, Genic enhancers	rs11571604
BRCA2	c.8633-1274A>G	Benign	Intron 20	1	BRCAexchange/IARC	HSF, SRY, MYB, stat4,GR-beta	TF binding region Chromatin interactive region	rs150600452
BRCA2	c.317-512A>T	Uncertain Significance	Intron 3	1		SRY, NKX25, NRSF, C-MYC	Chromatin interaction	
CHEK2	c.1505-13A>G	Uncertain Significance	Intron 13	1		CUTL1 , NFY ,FOXI1	TF binding region	rs1064793330
NBN	c.995-3C>T	Uncertain Significance	Intron 8	1		TST1, OCT1,CDXA, GR-alpha	Splicing region - non impatto sullo splicing (Human Splicing Finder)	
RAD50	c.3475+41C>G	Uncertain Significance	Intron 22	1		GR-alpha ,TFII-I,Pax-5	TF binding region Chromatin interaction	rs1483042111
RAD50	c.1052-23T>C	Benign	Intron 7	1	LOVD	GR-beta, HFH", CDXA, OCT1		rs200246462
STK11	c.*16+42G>A	Uncertain Significance	intron 9	1		TF binding region CpG island Chromatin interaction		rs934771316

e)

**Gene**	**Nt.change**	**Clinical Classification**	**Position**	**N.Patient**	**Database**	**Transcription factor**	**rVarBase/ENCODE**	**dbSNP**
ATM	c.8850+689A>C	Uncertain Significance	Intron 61	1	ClinVar Miner	MZF1, CP2, EBF (Tfbind)		rs189328374
ATM	c.8850+389G>A	Uncertain Significance	Intron 61	1		CUTL1, P300 , FACB,YY1, PAX-4A, POU3F2, PEX14 , MYOD(Tfbind)		

BRIP1	c.2379+3737A>C	Uncertain Significance	intron 16	1		GATA1,2,3, PAX5, XFD3, MZF1, CAP, CDPCR3HD, CMYB (Tfbind), YY1, T3R-beta1, FOXP3 (Promo), C/EBPbeta (Tf bind, Promo)	TF binding region, Chromatin interaction	rs781102554
RAD51C	c.404+63_404+71dupGTACTATCG	Uncertain Significance	Intron 2	1		C/EBPbeta, HNF-3alpha, TFIID , GATA1, GATA2(Tfbind)	TF binding region Chromatin interactive region	rs142735413
RAD51D	c.144+558C>T	Uncertain Significance	Intron 2	1		GATA3, NRSF((Tfbind)	TF binding region,Chromatin interactive region, Genic enhancers, Active TSS, Flanking Active TSS	
TP53	c.-29+502delC	Uncertain Significance	intron 1	1		MYB, Elk-1, CETS1(Tfbind)	TF binding region, Chromatin interaction	
TP53	c.-2+1049C> G	Uncertain Significance	intron 1	1		MZF1**,** p300(Tfbind)	TF binding region, Chromatin interaction	rs939819655|
TP53	c.-29+622G>C	Likely Benign	intron 1	1		NFKB, GC, AHRARNT, E2F, AHR, SP1, AP2, LYF1 (Tfbind), E2F-1 (Promo)MF3, ZF5, C/ERPbeta, LF-A1, DP-1, E2F1, E47, NF1(Lasagna)	TF binding region, CpG island, Chromatin interactive region, Enhancers, Bivalent/Poised TSS	rs189184134
TP53	c.-29+475C>A	Uncertain Significance	Intron 1	1		NKX2, RAR-beta:RXR-alpha(Promo)	Bivalent/Poised TSS, ZNF genes & repeats, TF binding region, Chromatin interactive region	

Table S2: List of 75 non-coding variants identified in 5'UTRs (a), 3'UTRs (b), splice regions(c), intronic regions(d), and the incidental findings (e). The table reports the nucleotide change for each variant, their clinical classification (P = Pathogenic, LP = Likely-pathogenic, VUS = Variant of Unknown clinical Significance, LB = Likely-benign), their frequency in patients analyzed, the chromatin states of surrounding regions (rVarBase/ENCODE), any registration in databases and the variant dbSNP code. The predictions on putative TF binding sites were evaluated by *Promo*, *TF Bind* and *Lasagna* software **(a, d, e)**, the presence of consensus sequences for miRNA binding sites was analyzed *by TargetScanHuman* and *miRDB* **(b)** and the investigation on potential splice sites was performed by *Human Spicing Finder* **(c).**

a)

Genomic regions	Genomic localization	Length (bases)
BRCA1 promoter	chr17: 41,277,500 – 41,278,500	1000
BRCA1 5’UTR (exon1A)	chr17: 41,277,287 – 41,277,500	223
BRCA1 5’UTR (exon 1B)	chr17: 41,277,340 – 41,277,197	145
BRCA1 5’UTR (exon 2 to ATG)	chr17: 41,276,110 – 41,276,133	22
BRCA1 Intron 2 (region 1)	chr17: 41,271,250 - 41,272,100	850
BRCA1 Intron 2 (region 2)	chr17: 41,275,493 - 41,276,019	526
BRCA1 intron 12 (region 1)	chr17: 41,237,500 – 41,237,850	350
BRCA1 intron 12 (region 2)	chr17: 41,236,600 – 41,236,960	360
BRCA1 Intron 13	chr17: 41,231,460 - 41,232,155	695
BRCA1 intron 16	chr17: 41,220,900 – 41,221,250	350
BRCA1 3’UTR (exon 24)	chr17: 41,196,311 – 41,197,698	1387
BRCA2 promoter	chr13: 32,888,616 – 32,889,616	1000
BRCA2 5’UTR (exon 1)	chr13: 32,889,616 – 32,889,805	189
BRCA2 5’UTR (exon 2 to ATG)	chr13: 32,890,558 – 32,890,600	42
BRCA2 Intron 3	chr13: 32,898,060 - 32,898,621	561
BRCA2 Intron 8	chr13: 32,904,481 - 32,905,003	522
BRCA2 Intron 24 (region 1)	chr13: 32,955,507 - 32,956,161	654
BRCA2 Intron 24 (region 2)	chr13: 32,958,773 - 32,959,472	699
BRCA2 Intron 24 (region 3)	chr13: 32,964,590 - 32,965,788	1198
BRCA2 3’UTR	chr13: 32,972,904 – 32,973,809	905

b)

Genomic regions	Genomic localization	Length (bases)
PALB2 5’UTR	chr16: 23,652,480 -23,652,715	235
PALB2 3’UTR	chr16: 23,652,491 -23,652,782	291
PTEN 5’UTR	chr10: 89,622,882 -89,624,235	1354
PTEN 3’UTR	chr10: 89,725,231 -89,731,706	6475
TP53 5’UTR	chr17: 7,590,696 -7,590,860	164
TP53 3’UTR	chr17: 7,571,723 -7,572,936	1213
RAD51C 5’UTR	chr17: 56,769,568-56,770,006	438
RAD51C 3’UTR	chr17: 56,811,586-56,811,710	124
RAD51D 5’UTR	chr17: 33,446,642 -33,448,545	1903
RAD51D 3’UTR	chr17: 33,427 ,692 -33,427,981	289
ATM 5’UTR	chr11:108,093,562 -108,098,352	4790
ATM 3’UTR	chr11:108,236,234 -108,239,837	3603
CDH1 5’UTR	chr16: 68,771,129 -68,771,320	191
CDH1 3’UTR	chr16: 68,867,415 -68,869,491	2076
CHEK2 5’UTR	chr22: 29,137,758-29,137,828	70
CHEK2 3’UTR	chr22: 29,083,750-29,083,887	137
STK11 5’UTR	chr19: 1,205,743 -1,206,918	1175
STK11 3’UTR	chr19: 1,227,566 -1,228,450	884
BRIP1 5’UTR	chr17:59,940,652 -59,940,898	246
BRIP1 3’UTR	chr17:59,756,503 -59,760,684	4181
RAD50 5’UTR	chr5: 131,892,637 -131,893,020	383
RAD50 3’UTR	chr5: 131,978,078 -131,979,770	1692
NBN 5’UTR	chr8: 90,996,794 -90,996,951	157
NBN 3’UTR	chr8: 90,945,564 -90,947,864	2300

Table S3: Genomic coordinates of the regulatory regions covered with the probe panel a) of the 5'UTR, the 3'UTR and of the intronic regions of the *BRCA1/2*. **b)** of the 5’UTR and the 3’UTR of the 12 genes analyzed and the relative length covered in base pairs.
